# Pre-Anodized Graphite Pencil Electrode Coated with a Poly(Thionine) Film for Simultaneous Sensing of 3-Nitrophenol and 4-Nitrophenol in Environmental Water Samples

**DOI:** 10.3390/s22031151

**Published:** 2022-02-02

**Authors:** Vijaya Gopalan Sree, Jung Inn Sohn, Hyunsik Im

**Affiliations:** Division of Physics and Semiconductor Science, Dongguk University, Seoul 04620, Korea; junginn.sohn@dongguk.edu (J.I.S.); hyunsik7@dongguk.edu (H.I.)

**Keywords:** pencil graphite electrode, pre-anodization, poly(thionine), 3-nitrophenol, 4-nitrophenol, water samples

## Abstract

A very simple, as well as sensitive and selective, sensing protocol was developed on a pre-anodized graphite pencil electrode surface coated using poly(thionine) (APGE/PTH). The poly(thionine) coated graphite pencil was then used for simultaneous sensing of 3-nitrophenol (3-NP) and 4-nitrophenol (4-NP). The poly(thionine) coated electrode exhibited an enhanced electrocatalytic property towards nitrophenol (3-NP and 4-NP) reduction. Redox peak potential and current of both nitrophenols were found well resolved and their simultaneous analysis was studied. Under optimized experimental conditions, APGE/PTH showed a long linear concentration range from 20 to 230 nM and 15 nM to 280 nM with a calculated limit of detection (LOD) of 4.5 and 4 nM and a sensitivity of 22.45 µA/nM and 27.12 µA/nM for 3-NP and 4-NP, respectively. Real sample analysis using the prepared sensor was tested with different environmental water samples and the sensors exhibited excellent recovery results in the range from 98.16 to 103.43%. Finally, the sensor exposed an promising selectivity, stability, and reproducibility towards sensing of 3-NP and 4-NP.

## 1. Introduction

The U.S environmental protection agency (USEPA) classified that the nitroaromatic compounds (2-nitrophenol, 3-nitrophenol, and 4-nitrophenol) are environmentally hazardous materials due to their harmful nature, where even low-level concentrations can affect living organisms including humans, animals, and plants [1,2,3,4]. When these nitrophenol isomers enter the human body, they can cause several health problems such as headaches, drowsiness, fever, and methemoglobinemia [5,6]. Moreover, these compounds severely affect plants’ growth through water contamination (0.7 mM/L of nitrophenols) [7]. However, huge quantities of nitrophenols are used in various industries such as pharmaceuticals, pesticides, dyes, and explosives [8,9,10], leading to nitrophenol isomers being present not just in water sources but also in soil samples. In particular, nitrophenols are largely found in different kinds of environmental water samples due to their easy solubility and high stability in aqueous media. These compounds are degradable due to the presence of strong electron-withdrawing species which produce an unpleasant smell and bitter taste, thus greatly affecting the environment of aquatic living species [11,12,13]. Therefore, the maximum allowed concentration level of these nitrophenols in the drinking water is 0.5–5 µM/L [14,15]. Nitrophenol isomers constantly react and influence other phenolic compounds due to their structural similarity and physio-chemical nature [16,17]. Hence, sensing of these nitrophenol isomers in environmental water samples is an essential task.

Different analytical methods such as spectrometry [18,19], high-performance liquid chromatography with different detectors [20,21], capillary electrophoresis [22], flow injection detection [23], fluorescence [24,25], surface-enhanced Raman spectroscopy (SERS) [26], and chemiluminescence [27,28] have been used to quantify these nitrophenol isomers. However, common analysis procedures take a long time and are highly expensive, requiring sophisticated instrumentation facility with samples that were pre-treated [29]. Among these techniques, the electrochemical analysis has been considered an outstanding and alternative method for nitrophenol quantification, several advantages such as an easy method of sample preparation, simple and continuous steps for sample analysis, and rapid output response [30,31]. Moreover, these analytical instruments are portable and low-cost, making them uncomplicated and suitable for onsite monitoring.

Recently, different kinds of nanomaterials and conductive polymer-based modified materials have been used for simultaneous and individual sensing of nitrophenol isomers [32,33]. Nanomaterials and polymers improve the electrocatalytic property as well as the surface area of the modified electrodes to achieve high sensitivity and selectivity of the nitrophenols [34,35]. Compared to the nanomaterial-based modified materials, polymer-based materials have massive advantages recently due to their high stability, interlinking the different active sides [35], and uniformity during the electrochemical deposition/polymerization with robust adherence on the electrode surface [36]. Electropolymerization/deposition is highly advantageous in securing the polymer on the electrode surface. The polymer film width, pervasion, and charge transfer characteristic can be controlled simply by tuning the electroanalytical optimization conditions [37,38]. The highly redox-active polymer of poly(thionine) has been coated on different kinds of the electrode surfaces and then used in various electrochemical sensing applications such as pre-treated glassy carbon electrode (GCE) coated with poly(thionine) for sensitive detection of dopamine [39], poly(thionine)/gold composite coated on ITO electrode for label-free detection of DNA hybridization [40], poly(thionine)/CuO nanoparticles coated pencil for paracetamol sensing in the presence of ascorbic acid [41], poly(thionine) coated on a carbon screen-printed electrode for flow-injection method analysis of NADH [42], and carbon nanotube/poly(thionine) modified carbon film electrode for simultaneous sensing of acetaminophen and dipyrone [43]. Poly(thionine) has exhibited excellent electrocatalytic activity in the detection of neurotransmitters, hydroxy benzenes, and other biologically important molecules [44,45,46]. However, poly(thionine) used for hydrogen peroxide reduction in different food and cosmetic samples has also been reported [47,48]. Herein, a new analytical protocol for poly(thionine) formation on pre-anodized pencil graphite electrodes is developed to show the enhanced electrocatalytic reduction of 3-nitrophenol and 4-nitrophenol. However, the 2-nitrophenols were not detected due to their intramolecular hydrogen bonding through the -OH and -NO_2_ moieties that were present in the nearby position at the benzene ring [49,50]. Therefore, APGE/PTH has been utilized for the electrocatalytic sensing of 3-NP and 4-NP and provided an excellent electrochemical response in different environmental water samples.

In this work, poly(thionine) (PTH) coated on a pre-anodized graphite pencil electrode (APGE) was used to study the electrocatalytic behavior towards selective reduction of 3-NP and 4-NP. The APGE/PTH has shown that these two nitrophenols were individually and effectively separated and, their simultaneous sensing was also tested. The proposed sensing material has shown an excellent catalytic activity, high sensitivity, well-defined peak separations, long-term stability, and reproducibility.

## 2. Materials and Methods

### 2.1. Chemicals and Reagents

The analytical grade of chemicals such as 2-nitrophenol, 3-nitrophenol, 4-nitrophenols, thionine, catechol, K_2_HPO_4_, and KH_2_PO_4_ were bought from Sigma-Aldrich (USA). Other reagents purchased were of analytical reagent quality without extra purification. Phosphate buffer saline (0.1 M, PBS) was prepared by mixing K_2_HPO_4_ and KH_2_PO_4_ solutions followed by altering the pH with sodium hydroxide (NaOH) or phosphoric acid (H_3_PO_4_). All the target analytes’ (3-NP and 4-NP) stack solutions (0.01 mM) were prepared with deionized water. All the stock solutions were placed in a black place to eliminate the direct contact of sunlight. All the aqueous solutions used for the electrochemical experiment were prepared using double distilled water that was collected from the Milli-Q water cleansing chamber (18.2 MΩ cm). All electrochemical experiments were carried out at room temperature (26 ± 1 °C) and purified inert nitrogen gas was purged in the electrolyte for 300 s before electrochemical measurements.

### 2.2. Instruments

The electroanalytical measurements were carried out using a CHI 660B potentiostat (CH Instruments Inc., Austin, TX, USA) with a portable three-electrode assembly. Pencil graphite (2.4 mm × 40 mm with disc area of 0.044 cm^2^) (PGE), pre-anodized pencil graphite electrode (APGE), and pre-anodized pencil graphite electrode on the poly(thionine) (APGE/PTH) electrode were used for the working electrodes and, platinum wire and silver/silver chloride (3 M KCl) were used as auxiliary and reference electrodes, respectively. All potentials were measured using this reference electrode. Electrochemical impedance spectroscopy (EIS) measurements were carried out in the frequency range from 0.1 Hz to 100 kHz in 5 mmol/L of K_3/4_[Fe(CN)_6_] in 0.1 mol/L of KCl solution with the following EIS amplitude of 5 mV and AC bias of 0.26 V. The functional group of APGE and APGE/PTH electrodes were confirmed using FT-IR spectra obtained from JASCO FT-IR-4600 (Tokyo, Japan).

### 2.3. Fabrication of APGE and APGE/PTH

Four HB pencils were collected from the local stationery shop and the pencils’ wooden parts were removed with a sharp knife (Figure S1). The pencils’ graphite rods were cleaned with ethanol and water and were then allowed to dry. The graphite rods had some micropores and cavities. Hence, to prevent these micropores, paraffin wax was impregnated on the cavity surface following our previously reported method [51]. In brief, one end of pencil graphite rod surface was polished with different qualities (500 and 600 grade) of the emery paper, and finally, the smoothed surface was washed with ethanol and deionized water. The cleaned graphite rods were kept in an electrochemical cell comprising 0.1 M PBS followed by pre-anodization by applying 1.8 V for 300 s on the working electrode. APGE/PTH was fabricated and measured by sweeping the potential from −0.4 to 0.1 V with a scan rate of 50 mV/s in 0.1 M PBS containing 0.5 mmol/L of thionine (Figure S2). PTH coated on APGE was washed with deionized water to remove unreacted thionine monomers. Finally, a dark blue color was observed which might be due to the PTH on the surface of the electrode [39].

## 3. Results and Discussion

### 3.1. Surface Morphology and FT-IR Measurements of APGE and APGE/PTH

**Figure 1:**
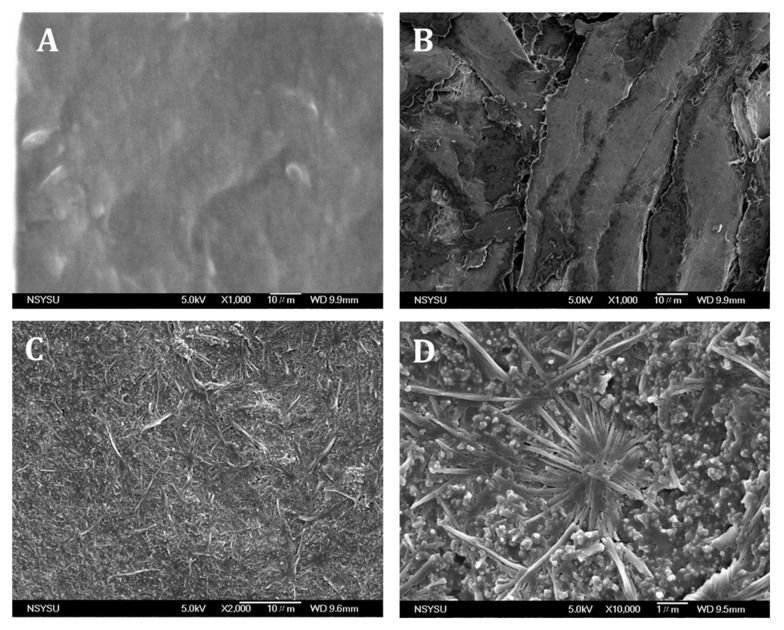
SEM images of (**A**) pencil graphite rod, (**B**) Pre-anodized pencil graphite rod, (**C**,**D**) Poly(thionine) on the pre-anodized pencil graphite rod with different magnifications.

**Figure 2:**
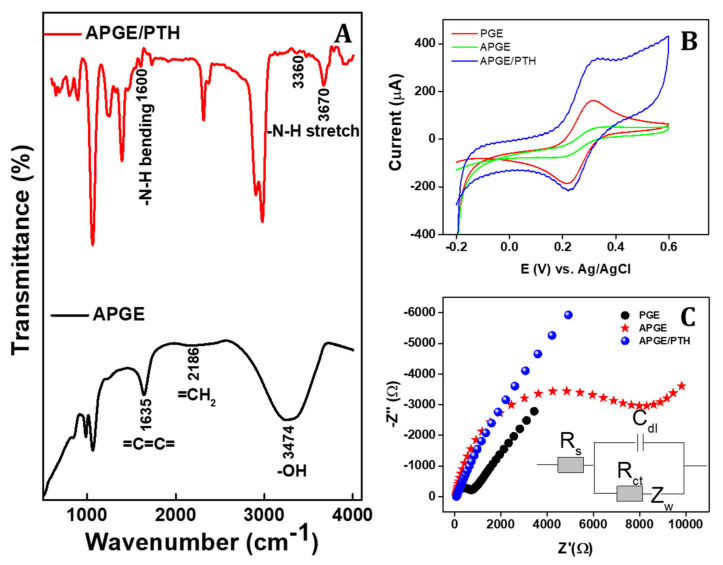
(**A**) FT-IR spectra of the APGE and APGE/PTH, (**B**) electrochemical CV response of different electrodes in 5 mmol/L of K_3/4_[Fe(CN)_6_] in 0.1 M KCl with scan rate of 50 mV/s and (**C**) EIS response of the different electrodes in 5 mmol/L of K_3/4_[Fe(CN)_6_] in 0.1 M KCl at Eapp is 0.18 V vs. Ag/AgCl (inset figure, Randles equivalent circuit).

The scanning electron microscopic images of the pencil graphite rod, pre-anodized pencil graphite rod, and poly(thionine)-coated pencil graphite rod electrodes are illustrated in Figure 1. A typical rough surface was observed on the pencil graphite rod (Figure 1A). The pre-anodized graphite pencil rod exhibited mesoporous flack-type morphology and the morphological changes confirm that the -OH and -COOH groups were attached on the surface of the graphite rod (Figure 1B). From Figure 1C,D, it is clear that the graphite flacks were covered with a polymer film (the rod-like structures) confirming the film formation on the electrode surface. Hence, these surface morphological studies confirm that poly(thionine) was directly linked on the graphite electrode surface. Previously, it has been reported that the preanodization of carbon electrodes creates oxygen rich functional groups, such as carbonyl, phenolic, alcoholic, carboxylic and ether, on its outer surface and further helps the promotion of the electron oxidation/reduction of certain small molecules and bio-chemicals [52]. To confirm the surface functional groups, APGE and APGE/PTH were studied by FT-IR spectral method. Figure 2A shows that the APGE electrode has broad stretching vibration of -OH at 3274 cm^−1^, sp^2^ carbon (=CH=CH=) stretching vibrations (breathing mode) at 1635 cm^−1^ and the symmetric and unsymmetric stretching mode of =CH_2_ at 2186 cm^−1^ [52]. Further, PTH coated on the APGE surface showed significant vibration for primary amine (-N-H) stretching at 3670, 2990, 2900, and 3360 cm^−1^ followed by 1600 cm^−1^ corresponding to the C-S bond stretching [53]. Hence, FT-IR spectral studies confirmed that the -OH and -COO- moieties were present on the APGE surface. Additionally, electrochemical polymerization of PTH on the APGE surface was also confirmed.

### 3.2. Electrochemical Characterization of APGE/PTH

The surface characteristics of the fabricated APGE and APGE/PTH were studied using the CV and EIS methods. Figure 2B illustrates CV responses of the electrodes PGE, APGE, and APGE/PTH in 5 mmol/L of K_3/4_[Fe(CN)_6_] 0.1 M of KCl at a scan rate of 50 mV/s. The peak-to-peak separation of the three electrodes was estimated to be 0.10, 0.14, and 0.07 mV, corresponding to PGE, APGE, and APGE/PTH, respectively. APGE showed broad redox peaks with lower current response in the K_3/4_[Fe(CN)_6_] electrolyte, confirming that the electrode surface has negatively charged species such as hydroxyl and carboxyl groups. These negatively charged groups undergo electrostatic repulsion between the electrode surface and electrolyte solution [39]. Furthermore, with the modified PTH on the APGE surface, the redox peak current was enhanced and a lower ∆Ep value was achieved. The lower ∆Ep indicates that APGE/PTH has a facetious electron movement property of the electrode. Based on Randles-Sevick equation [54],

ipc=2.69×105 Ae D1/2 n3/2 ν1/2 c

where *n* represents the quantity of electron transfer in the redox couple, *D* represents the diffusion coefficient (7.6 × 10^−6^ cm^2^ s^−1^), *c* represents the concentration of the redox couple, and *Ae* represents the electroactive available surface area. Here, Ae was estimated to be 0.0732, 0.0976, and 0.1235 cm^2^ corresponding to the PGE, APGE, and APGE/PTH, respectively. At the same time, electrochemical impedance spectral (EIS) measurements were studied in 0.1 M KCl containing 5 mmol/L of K_3/4_[Fe(CN)_6_] as a redox probe with a bias voltage of 0.19 V vs. Ag/AgCl. Figure 2C shows the EIS response of (Nyquist graph of Z′ vs. −Z″) PGE, APGE, and APGE/PTH. All the obtained EIS data were fitted with Randle’s equivalent circuit (Figure 1C-inset). From the Randle’s equivalent circuit, charge transfer resistance (*R_ct_*), and Warburg impedance (Z_w_) are side by side to double layer capacitance (C_dl_). The half-circle diameter is matched with the *R_ct_* value which indicates that the electron transfer process in the redox species is in the interface between the electrode and electrolyte [52]. PGE in the Nyquist graph form showed a semicircle with an *R_ct_* of 750 Ω, and APGE showed large semicircle with an *R_ct_* of 8000 Ω. This large semicircle is due to the repulsion between the pre-anodized electrode that has negatively charged hydroxyl and carboxyl groups that undergoes electrostatic repulsion between the electrolyte and electrode. At the same time APGE/PTH showed a lower semicircle with an *R_ct_* of 180 Ω. Based on the *R_ct_* values, the switched current density (*jo*) was calculated for all the three different kinds of electrodes using the following equation [55]

$$jo=RT/nFAR_{ct}$$

The *R* represents gas constant (8.314 J/K/mol); *T,* the ambient temperature (298 K); *n,* the quantity of electrons transferred through the redox reaction (1); *F*, the Faraday constant (96,485 C/mol); and *A,* the surface area of the electrode (0.04 cm^2^). The obtained *jo* values were 0.0123, 0.0032, and 0.2128 A cm^−2^ for PGE, APGE, and APGE/PTH, respectively. Compared to other electrodes, the APGE/PTH coated electrode showed an almost three-times-higher value. Hence, with lowest obtained *R_ct_* (EIS response), as well as higher *jo* values, it is clear that APGE/PTH exhibits excellent electrochemical behaviors that are advantageous for the electrochemical experiments [55,56,57].

### 3.3. Optimization of PTH on the APGE towards Sensing of 3-NP and 4-NP

**Figure 3:**
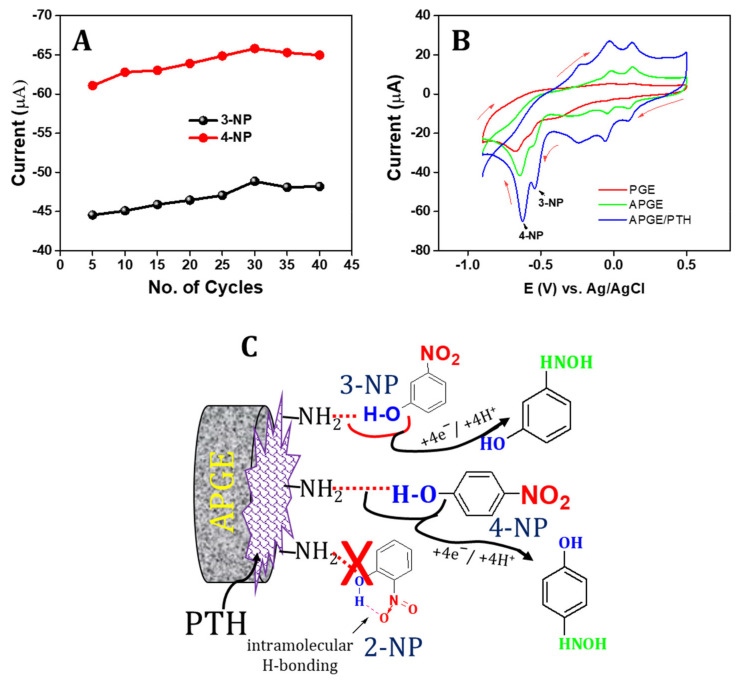
(**A**) The cathodic peak current dependence of 3-NP and 4-NP (20 µM of each) on the number of cycles of the cyclic voltammogram during the thionine electrochemical polymerization, (**B**) Cyclic voltametric response of the different modified electrodes in 0.1 M PBS containing 20 µM of 3-NP and 4-NP at scan rate of 50 mV/s and (**C**) Schematic representation of the proposed mechanism for sensing of 3-NP and 4-NP at APGE/PTH via hydrogen bond formation.

A different series of cyclic voltammograms were studied for the optimization of poly(thionine) formation on the APGE surface. The poly(thionine) thickness was directly connected with the number of sweeping segments throughout the electrochemical polymerization [39]. The poly(thionine) film thickness was thus studied by numerous polymerization cycles on the sensing of 3-NP and 4-NP. The PTH was prepared via the electrochemical polymerization technique on the pre-anodized graphite electrode in 0.1 M PBS in 0.5 mmol/L of thionine and a cycling range from 5 to 40 with a scan rate of 50 mV/s. Figure 3A shows the different forms of electrocatalytic behavior of PTH with a different number of sweeping cycles. The redox peak and cathodic current were increased after increasing the number of sweeping segments up to 30 cycles. After 30 cycles, the cathodic peak current decreased slightly. This behavior demonstrates the activity of PTH at certain thickness levels and electron transfer of the polymer thickness barrier [58]. Hence, 30 sweeping cycles was chosen as the optimum condition for the electrocatalytic sensing of 3-NP and 4-NP.

### 3.4. Electrocatalytic Behavior of APGE/PTH towards Sensing of 3-NP and 4-NP

The electrocatalytic behavior of the fabricated APGE/PTH electrode towards the sensing of 3-NP and 4-NP was studied using cyclic voltammetry. Figure 3B shows cyclic voltammetry results for 0.1 mmol/L of 3-NP and 4-NP for unmodified PGE, APGE, and APGE/PTH modified electrodes. For the unmodified PGE, the redox peaks of 3-NP and 4-NP were observed at 0.13/0.09 V and −0.01/−0.04 V with reduction potentials of −0.58 and −0.60 V, respectively. The calculated peak-to-peak separations (∆Ep) were 0.04 V and 0.03 V, and nitrophenol isomers’ reduction potential was measured at −0.58 and −0.60 V for 3-NP and 4-NP, respectively. The APGE exhibited redox peaks at 0.12/0.10 V and −0.01/−0.03 V with a calculated ∆Ep of 0.02 V and 0.02 V. In addition, the reduction of nitrophenols was observed at −0.55 and −0.64 V for 3-NP and 4-NP, respectively. Finally, APGE/PTH coated electrode redox potential was measured at 0.07/0.08 V and −0.04/−0.05 V with calculated ∆Ep of 0.01 and 0.01 V, respectively. Reduction of these nitrophenols was observed in the potential of −0.52 and −0.61 V for 3-NP and 4-NP, respectively. This cyclic voltammetry result indicates that redox reactions were reversible for both APGE and APGE/PTH while the unmodified PGE was quasi-reversible towards sensing of 3-NP and 4-NP. However, compared to other electrodes, the APGE/PTH coated electrode showed excellent electrocatalytic behavior towards sensing of 3-NP and 4-NP.

### 3.5. Electrocatalytic Mechanism towards 3-NP and 4-NP

The electrocatalytic sensing of 3-NP and 4-NP for the voltammetric signal peak-to- peak separation can be explained by different routes. Initially, PTH behaves as an electron transfer mediating material [59] to improve electron transfer kinetics for the catalytic reduction of 3-NP and 4-NP by following

$$3–\mathrm{NP}\;\left(o\right)\;+\;\;\mathrm{PTH}\;\left(r\right)\;\to \;3–\mathrm{NP}\;\left(r\right)\;+\;\;\mathrm{PTH}\;\left(o\right)$$

$$4–\mathrm{NP}\;\left(o\right)\;+\;\;\mathrm{PTH}\;\left(r\right)\;\to \;4–\mathrm{NP}\;\left(r\right)\;+\;\;\mathrm{PTH}\;\left(o\right)$$

While the potential cycling is in the negative direction, the PTH has reduced and become catalytically active to reduce 3-NP and 4-NP. In the next step, PTH has untied NH_2_ moiety which can make hydrogen linkage with hydroxides moieties of 3-NP and 4-NP. This step significantly donates flagging the -OH linkage to enable the electron transfer via O-H-N bond formation [60]. Figure 3C illustrates the sensing mechanism of 3-NP and 4-NP via hydrogen linkage. It clearly shows that the particular electron transfer kinetics are quite equal for 3-NP and 4-NP due to their different skeletal properties. While, 3-NP and 4-NP -OH groups easily participate in hydrogen bonding, it is not relevant for 2-NP, as 2-NP undergoes intramolecular hydrogen bonding through -OH and NO_2_ groups. Therefore, the peak currents of these two nitrophenol isomers exhibit higher catalytic current. The enhanced catalytic nature for the simultaneous sensing of 3-NP and 4-NP was accredited partially to the electrostatic interactivity within the surface of PTH and nitrophenol isomers with dissimilar pKa values. The 3-NP (pKa = 8.36) and 4-NP (pKa = 7.15) isomers existed in the form of protonation at pH-7.

### 3.6. pH Influence

**Figure 4:**
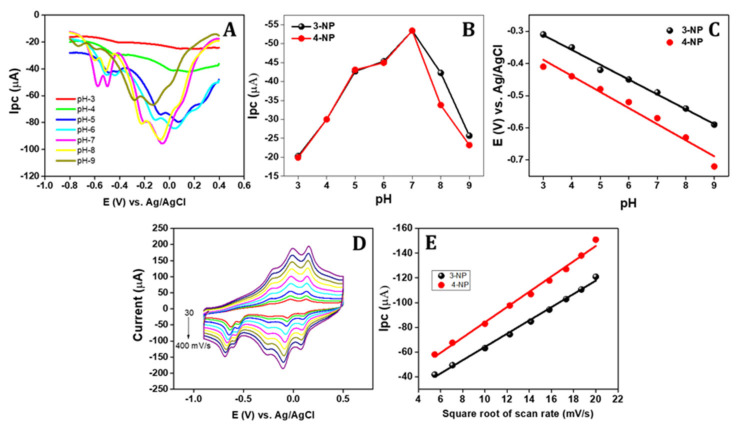
(**A**) Influence of different pH condition (pH-3 to pH-9) on sensing (20 µM) of 3-NP and 4-NP; (**B**) the relationship between pH vs. cathodic peak current of both 3-NP and 4-NP; (**C**) the relationship between pH vs. cathodic potential shift of both 3-NP and 4-NP; (**D**) the influence of different scan rates (30 to 400 mV/s) in the presence of 20 µM of 3-NP and 4-NP in 0.1 M PBS; and (**E**) the relationship between the square root of scan rate vs. cathodic peak current of 3-NP and 4-NP.

The influence of the electrolyte pH on the sensing of 3-NP and 4-NP using APGE/PTH was studied in the pH range from 3 to 9, and the obtained voltammetric outputs are displayed in Figure 4A. The cathodic currents of 3-NP and 4-NP were raised slowly with the increase in pH from 3 to 7 (Figure 4B). Over pH 7, the cathodic current gradually reduced in the pH range from 8 to 9. This cathodic peak current decreases due to the protonated form of the amine group (-NH_3_^+^) of nitrogen on the PTH at lower pH [37]. While increasing the pH, the creation of NH_3_^+^ gradually decreases and excess protic aromatic compounds are produced. The 3-NP and 4-NP can connect with the nitrogen atoms present on the PTH polymer. However, the cathodic peak current was enhanced with the higher pH condition from 3 to 7, whereas at neutral pH (PBS-7), 3-NP and 4-NP become the deprotonated form of anions, and they are ready for the reduction process [12]. Meanwhile, PTH containing nitrogen atoms may become deprotonated and produce negative charge at high pH conditions. Hence, the electrostatic repulsion between the target 3-NP/4-NP and the surface of the electrode might be one of the main reasons for the decrease in the peak current of these analytes in the pH range from 8 to 9. In addition, the relation between pH and the cathodic potential of 3-NP and 4-NP was studied and the results are shown in Figure 4C. In Figure 4C, the cathodic peak potential has shifted towards the cathodic direction in the pH range from 3 to 9 for both analytes. The linear regression equation of the cathodic peak potential with the pH for these analytes are Epc = −57 pH + 367 mV (R^2^ = 0.9926) and Epc = −58 pH + 229 mV (R^2^ = 0.9963) for 3-NP and 4-NP, respectively. Both calibration lines were almost equal, which might explain why the peak potential of 3-NP and 4-NP are almost constant. From the calibration plots, slope values from the equation suggest that the electron transfer on the surface of the electrode is an equal number of protons that were coupled with an equal number of electrons that were transferred [12]. Hence, to attain high sensitivity, pH-7 (PBS) was chosen for simultaneous sensing of 3-NP and 4-NP.

### 3.7. Scan Rate Influence

The scan rate influence on the electrocatalytic sensing of 3-NP and 4-NP were studied using the APGE/PTH electrode. From Figure 4D, the reduction peak potential of 3-NP and 4-NP were shifted to the positive side, even while increasing the scan rate from 30 to 400 mV/s. The cathodic current of 3-NP and 4-NP increased with the increasing scan rate. The cathodic peak current of these analytes was directly proportional to the square root of scan rate (Figure 4E) and the linear regression equation is Epc = −5.35 V + 10.55 (R^2^ = 0.9957) and Epc = −6.17 V + 22.39 (R^2^ = 0.9927) for 3-NP and 4-NP, respectively. Hence, these results suggest that the electrode reaction is a diffusion-controlled process [34,61].

No absorption of 3-NP and 4-NP on the APGE/PTH electrode was observed. To confirm this phenomenon, the cyclic voltammetry for the 3-NP and 4-NP sensing in PBS solution at successful cycles was studied. The voltammogram results show that the cathodic peak current remained constant while repeating the cyclic voltammograms. These results clearly suggest that there is no absorption of 3-NP and 4-NP on the surface of the electrode.

### 3.8. Simultaneous DPV Method Sensing of 3-NP and 4-NP

**Figure 5:**
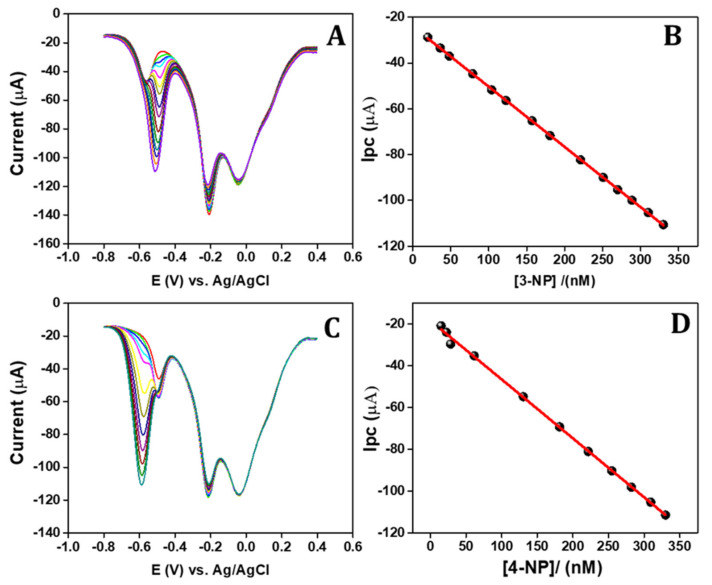
(**A**) DPV response of the APGE/PTH in different concentrations of 3-NP in the presence of 20 nM of 4-NP; (**B**) 3-NP concentration vs. cathodic peak current calibration plot; (**C**) DPV response of 4-NP at different concentrations in the presence of 20 nM of 3-NP; and (**D**) 4-NP concentration vs. cathodic peak current calibration plot.

**Figure 6:**
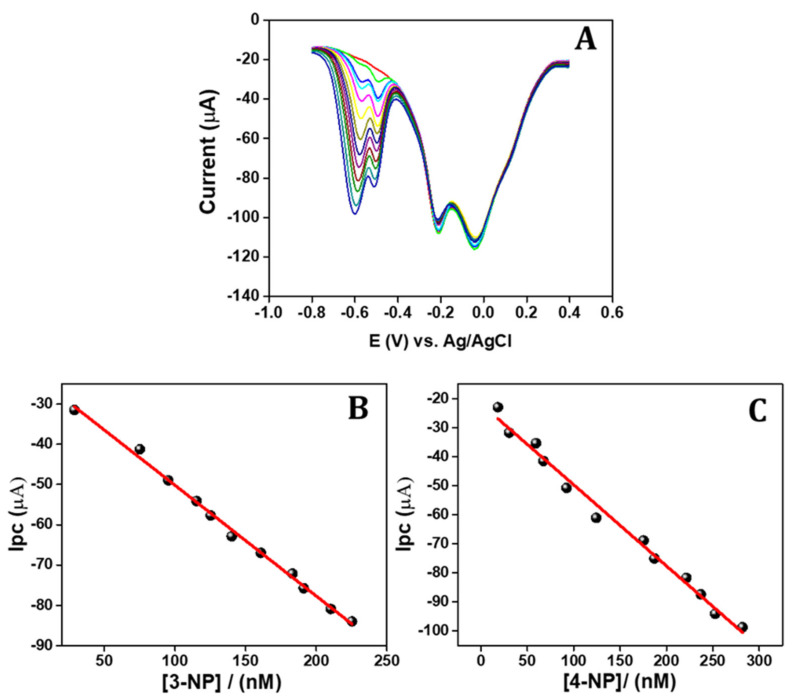
(**A**) DPV response of simultaneous sensing of 3-NP and 4-NP in various concentrations at 0.1 M PBS and (**B**,**C**) 3-NP and 4-NP concentration and corresponding cathodic current response calibration plot.

Based on the previous experimental results, the APGE/PTH electrode is proposed for the sensing of 3-NP and 4-NP, which can be detected independently and simultaneously via the differential pulse voltammetry technique. Figure 5A illustrates the differential pulse voltammogram of the different quantities of nitrophenol isomers. From the voltammograms it can be seen that the reduction potential of 3-NP sensing is at −0.47 V and the cathodic current increases linearly with the increasing concentration of 3-NP. Figure 5B shows the obtained linear concentration ranges from 20 to 330 nM with the linear regression equation of *ipc* = −23.99 (3-NP) + 0.263 (R^2^ = 0.999) and a calculated LOD (S/N = 3) of 5 nM, with the reduction potential of 4-NP, measured at −0.56 V, remaining significantly unchanged. A similar procedure was followed for 4-NP detection and the results are displayed in Figure 5C. The obtained wide concentrations ranging from 15 to 330 nM with linear regression equation of *ipc* = −18.35 (4-NP) + 0.282 (R^2^ = 0.998) and a calculated LOD of 4 nM, without any interference with 3-NP (Figure 5D). The simultaneous sensing of these two nitrophenol isomers was tested and the results are displayed in Figure 6A. The DPV responses of both 3-NP and 4-NP were sensed simultaneously in concentrations ranging from 20 to 230 nM and 15 to 280 nM (Figure 6B,C) with *ipc* = −22.67 (3-NP) + 0.275 (R^2^ = 0.996) and *ipc* = −21.86 (4-NP) + 0.278 (R^2^ = 0.988), and calculated LODs of 4.5 nM and 4 nM and sensitivities of 22.45 µA/nM cm^2^ and 27.12 µA/nM cm^2^ for 3-NP and 4-NP, respectively. The analytical parameters of the developed sensor for simultaneous sensing of 3-NP and 4-NP were compared with results from earlier published works [1,15,17,34,62,63,64] and the comparison is displayed in Table 1.

### 3.9. Selectivity of the APGE/PTH

The common interference species for the simultaneous sensing of 3-NP and 4-NP were tested. Structurally similar molecules such as hydroquinone, resorcinol, 2-nitrophenol, phenol, and catechol were tested with the APGE/PTH electrode. No significant peak current was observed for resorcinol, catechol, hydroquinone, and phenol in the current operating potential range (0.4 to −0.8 V), and only a small hump was observed with 2-nitrophenol (200 µmol/L), the cathodic potential of which was measured at −0.42 V. This small hump might be due to the interaction between 2-nitrophenol and the electrode surface (repulsion). Therefore, these phenolic compounds’ coexistence does not affect the simultaneous sensing of 3-NP and 4-NP. Subsequently, the most common interfering ions, such as NH_4_^+^, Ca^2+^, Mg^2+^, Fe^2+^, NO^3-^, Zn^2+^, SO_4_^2-^, and citrate ions were tested at up to 100-fold excess concentration and no significant cathodic peak disturbances were observed. Hence, this interference study clearly showed that the simultaneous sensing of 3-NP and 4-NP is very reliable at ambient temperature and conditions.

### 3.10. Stability and Reproducibility of the APGE/PTH

Stability and reproducibility are significant key parameters of the proposed APGE/PTH sensing of 3-NP and 4-NP. The fabricated APGE/PTH electrode was studied by exposing it to different conditions to access its long-term performance and shelf life. The electrode was kept in an air-tight container at room temperature when not in use. The APGE/PTH electrode sensing of 3-NP and 4-NP in 0.1 M PBS was tested by parallel observation of the reduction current in each 3-day interval. The DPV response of the proposed electrode towards sensing of 3-NP and 4-NP in different intervals is shown in Figure S3. From the DPV response measured at different intervals, the proposed APGE/PTH showed stable current response while sensing 3-NP and 4-NP. The proposed electrode current response of these nitrophenol isomers retained 92.12% and 94.34% (from the initial current) after 60 days. These results indicate that the proposed electrode has high stability. Moreover, the proposed electrode was successfully examined for reproducibility by testing the six different electrodes under the same conditions. The RSD was 4.12% and 3.99% for 3-NP and 4-NP, respectively. The APGE/PTH electrode was stored in an air-tight container while not in use. Therefore, these analytical results suggest that the fabricated APGE/PTH electrode is a promising material for sensing nitrophenols with notable stability and reproducibility.

### 3.11. Real Sample Studies

The practical utility of the fabricated APGE/PTH for the simultaneous sensing of 3-NP and 4-NP in different environmental waters. Lab tap water, lake water, and river water samples were tested for the quantitative measurements. The standard addition method was used for the estimation of environmental water samples. The water samples were initially purified by using 0.45 µM cellulose filter paper followed by adjusting the pH of the filtered solution to pH-7 to match the PBS used earlier in the testing. Three similar measurements are studied for the detection of these nitrophenol isomers. All the obtained analytical results were collected and displayed in Table 2. The average recovery was found to be from 98.16% to 103.43%. These results indicate that this proposed protocol can be effectively used for the simultaneous sensing of 3-NP and 4-NP in environmental water samples.

## 4. Conclusions

A very sensitive, robust, and facile electroanalytical technique for the simultaneous sensing of 3-NP and 4-NP was developed using APGE/PTH. The modified electrode preparation steps were very simple compared with other electrode preparation protocols. APGE/PTH showed well-defined redox peaks in the sensing of 3-NP and 4-NP in cyclic voltammetry with a potential difference of 0.09 mV between 3-NP and 4-NP, a sufficient potential range for the simultaneous sensing of nitrophenol isomers. The possible sensing mechanism and the voltammetric signal separations of these 3-NP and 4-NP on the APGE/PTH have been discussed. The enhanced electrocatalytic property and reversible electron transfer of the redox reaction of 3-NP and 4-NP were achieved on the APGE/PTH electrode. Finally, the proposed electrode exhibited high sensitivity, with a low detection limit being achieved. Furthermore, the real-time utility of the fabricated sensor was tested in different environmental water samples with satisfactory recovery results.

## Figures and Tables

**Table 1:** Analytical parameters comparison studies of the APGE/PTH with other reported electrodes.

Modified Electrode	Technique	Analytical Parameter	3-NP	4-NP	Ref
APGE/PTH	DPV	Linear range (nmol/L)	20–230	15–280	Proposed work
		LOD (nmol/L)	4.5	4	
Polyfurfural/GCE	DPV	Linear range (mol/L)	750–100,000	750–100,000	[1]
		LOD (nmol/L)	50	40	
PSF/GCE	DPV	Linear range (nmol/L)	200–100,000	100–120,000	[17]
		LOD (nmol/L)			
(Fe_3_O_4_-Pt NPs)/GCE	DPV	Linear range (nmol/L)	100–1500	100–1500	[15]
		LOD (nmol/L)	45.3	48.2	
CalCOP-MPC/GCE	DPV	Linear range (nmol/L)	1000–400,000	1000–400,000	[34]
		LOD (nmol/L)	122	212	
SBCD-rGO/GCE	DPV	Linear range (nmol/L)	100–800,000	100–800,000	[17]
		LOD (nmol/L)	30	50	
OMCs/GCE	DPV	Linear range (nmol/L)	1000–100,000	2000–90,000	[62]
		LOD (nmol/L)	60	100	
PTTB/GCE	DPV	Linear range (nmol/L)	300–12,500	300–15,000	[63]
		LOD (nmol/L)	50	50	
Poly(p-ABSA)/GCE	DPV	Linear range (nmol/L)	3000–800,000	3000–800,000	[64]
		LOD (nmol/L)	500	300	

**Table 2:** Estimation of 3-NP and 4-NP in different environmental water samples.

Samples	Initial Conc.	Added (nmol/L)		Observed (nmol/L)		Recovery (%)		RSD (%)	
		3-NP	4-NP	3-NP	4-NP	3-NP	4-NP	3-NP	4-NP
Tap water	NS *	30.0	20.0	30.07 (±0.05)	19.89 (±0.03)	100.23	99.45	2.19	1.98
		50.0	50.0	49.78 (±0.08)	50.07 (±0.01)	99.56	100.14	2.32	2.14
		100.0	100.0	99.85 (±0.03)	100.08 (±0.07)	99.85	100.08	2.18	2.21
Lake water	NS *	30.0	20.0	30.09 (±0.08)	20.07 (±0.01)	100.30	100.35	1.87	1.56
		50.0	50.0	51.01 (±0.05)	49.08 (±0.08)	102.02	98.16	1.98	1.23
		100.0	100.0	99.03 (±0.01)	100.10 (±0.03)	99.03	100.10	3.23	3.18
River water	NS *	30.0	20.0	31.03 (±0.09)	20.08 (±0.03)	103.43	100.40	1.89	1.34
		50.0	50.0	49.96 (±0.05)	50.65 (±0.08)	99.92	101.30	2.33	2.12
		100.0	100.0	99.82 (±0.02)	100.08 (±0.07)	99.82	100.08	3.16	3.26

* NS—not sensing (below the detection limit)**.**

## Data Availability

Not applicable.

## Supplementary Materials

The following supporting information can be downloaded at: https://www.mdpi.com/article/10.3390/s22031151/s1. Figure S1: Photograph of a 4-HB graphite pencil, Figure S2: Electrochemical polymerization of thionine on APGE surface in 0.1 M PBS electrolyte at scan rate of 50 mV/s for 30 segments and Figure S3: Stability studies of APGE/PTH towards simultaneous sensing of 3-NP and 4-NP.
