# Supplementary material for: Pre-Anodized Graphite Pencil Electrode Coated with a Poly(Thionine) Film for Simultaneous Sensing of 3-Nitrophenol and 4-Nitrophenol in Environmental Water Samples
Source: Sensors (Basel). 2022 Feb 2;22(3):1151. doi: 10.3390/s22031151 (PMC8838205; doi:10.3390/s22031151)
Supplement: Supplementary file 1 [file sensors-22-01151-s001.zip › sensors-1570064-supplementary.pdf]

Electronic Supplementary Material

Pre-Anodized Graphite Pencil Electrode Coated on poly(thionine) Film for Simultaneous Sensing of 3-nitrophenol and 4-nitrophenol in Environmental Water Samples

Vijaya Gopalan Sree*, Jung Inn Sohn, Hyunsik Im

Division of Physics and Semiconductor Science, Dongguk University-Seoul, Seoul 04620, Republic of Korea.

Corresponding authors: *sreevg@dgu.ac.kr*

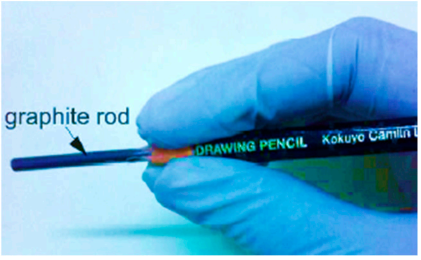

Figure S1. Photograph of a 4-HB graphite pencil

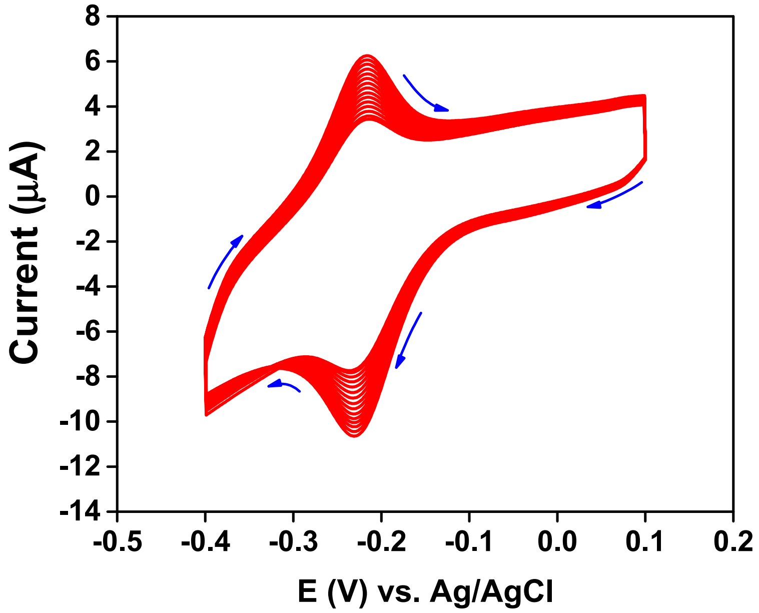

Figure S2. Electrochemical polymerization of thionine on APGE surface in 0.1 M PBS electrolyte at scan rate of 50 mV/s for 30 segments.

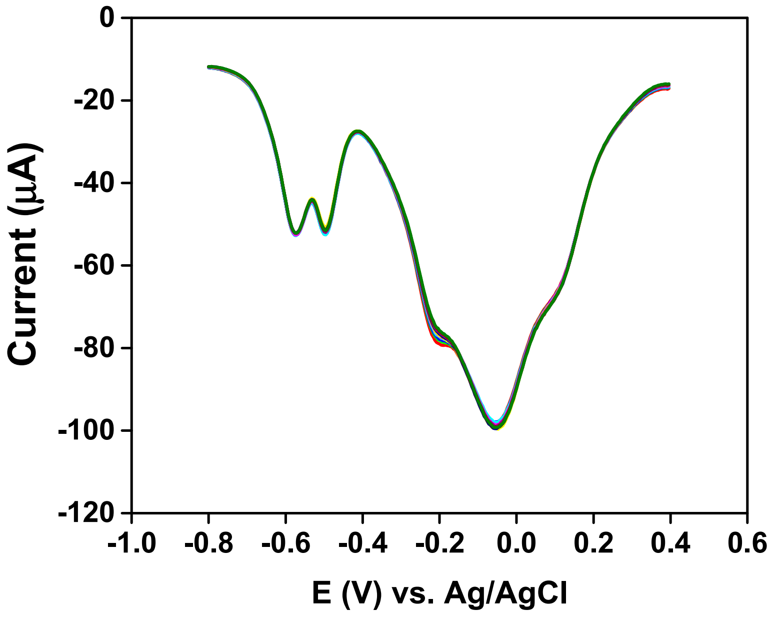

Figure S3. Stability studies of APGE/PTH towards simultaneous sensing of 3-NP and 4-NP
